# Relationship between obesity-related anthropometric indicators and cognitive function in Chinese suburb-dwelling older adults

**DOI:** 10.1371/journal.pone.0258922

**Published:** 2021-10-27

**Authors:** Weibo Ma, Hui Zhang, Ning Wu, Yuewen Liu, Peipei Han, Feng Wang, Jingru Wang, Fandi Xie, Shumeng Niu, Hao Hu, Chenyu Zhang, Nuo Chen, Yichen Zhang, Qi Guo, Ying Yu

**Affiliations:** 1 Department of Rehabilitation Medicine, Shanghai University of Medicine and Health Sciences, Shanghai, China; 2 Department of Economics and Management, East China Normal University, Shanghai, China; 3 Department of Rehabilitation Clinic, Shanghai Jiangwan Hospital, Shanghai, China; Medical University of Vienna, AUSTRIA

## Abstract

**Background:**

Studies relating obesity to cognition in older people show conflicting results, which may be explained by the choice of obesity indicators.

**Objectives:**

This study aimed to investigate the relationship between obesity-related indicators and cognitive impairment, especially between different age or gender subgroups, and explore whether obesity-related indicators were related to specific cognitive domains.

**Methods:**

This was a cross-sectional study on 1753 participants aged ≥ 60 years (41.0% men; aged 71.36 ± 5.96 years). Obesity-related indicators included body mass index (BMI), waist circumference (WC), calf circumference (CC), waist to hip ratio (WHR), waist to calf circumstance ratio (WCR), fat to fat-free mass ratio (FM/FFM). The Mini-Mental State Examination scale (MMSE) was used to assess cognitive function. Cognitive impairment was defined as a score ≤ 17 for illiterates, ≤ 20 for participants with primary school education, and ≤ 24 for those with junior high school degrees or above. Multiple logistic regression analysis was used to estimate multivariable-adjusted odds ratios (ORs) and 95% confidence intervals (CIs). Restricted cubic splines were used to analyze and visualize the linear relationships.

**Results:**

The prevalence of cognitive impairment was 18.77%. In the fully adjusted model, CC was negatively associated with cognitive impairment (OR = 0.94, 95% CI: 0.90−0.98). Further analysis showed that CC correlated positively with recall and place orientation. A higher FM/FFM was found to be associated with a higher prevalence of cognitive impairment (OR: 1.44, 95%CI: 0.88–2.35, *P* for trend = 0.029); this association was notable in women (*P* for trend = 0.002) and the oldest (*P* for trend = 0.009), and so did the potential effect of BMI on cognitive impairment (70–80 years: *P* for trend = 0.011; ≥ 80 years: *P* for trend = 0.013). No statistically significant association was found between cognitive impairment and WC, WHR, or WCR.

**Conclusion:**

CC and FM/FFM were associated with cognitive impairment in older people. Future research needs to distinguish the effects of fat and muscle mass on cognitive function, with special attention to different ages and genders.

## Introduction

Cognitive impairment is an age-related global disease that includes a spectrum from mild cognitive impairment to dementia. It can lead to enormous personal, community, and health care system costs [1]. By 2050, the proportion of people with dementia living in countries with low or middle incomes is expected to reach 71% [1]. Despite therapeutic advances, there is currently only limited success in treating this neurodegenerative disease. Therefore, identifying and managing modifiable risk factors for cognitive decline are imperative. Current research suggests that obesity may be one of the risk factors for cognitive impairment, as it may affect the brain through changes in structure, glucose metabolism, and inflammation [2, 3]. Obesity is an intervention-responsive factor, and related indicators are simple to measure and may function as tools for predicting cognitive decline. Therefore, the role of obesity in cognitive impairment is an important issue in preventing dementia in older people.

Research results on the effect of obesity on the cognition of older people are not consistent [3–6]. In recent decades, the "obesity paradox" had become particularly common among older people [7, 8]. This refers to the finding that obese people, when compared with people with normal weight and body mass index (BMI), have a lower risk of cognitive impairment, a better prognosis, and a lower mortality rate [9, 10]. The mechanism of the obesity paradox is largely due to better nutritional status and higher muscle retention [11]. In addition, adipokines, including leptin and insulin-like growth factors, can exert neuroprotective effects and suppress inflammatory responses [12]. In the absence of an explicit determinism of the mechanism linking obesity and cognition, we may be able to gain a more comprehensive and differentiated understanding of obesity by distinguishing between indicators of obesity heterogeneity.

Body mass index (BMI), waist circumference (WC), and waist-to-hip ratio (WHR) are generally used as diagnostic criteria for obesity in older people. The limitations of using traditional anthropometric measures have been well recognized: BMI as an indicator of general obesity can not distinguish between fat mass (FM) and fat-free mass (FFM) [13]; WC as an indicator of central obesity is difficult to rule out factors such as nutrition and muscle retention, and the WHR may mask the accumulation of abdominal fat if the hip circumference is also increased [14]. However, Waist to calf circumstance ratio (WCR) is an indicator to evaluate central obesity and nutrition and muscle retention at the same time, excluding the influence of nutrition and muscle retention. It also avoids the limitation of WC or WHR in response to central fat distribution [14, 15]. Furthermore, unlike general obesity and central obesity, calf circumference (CC), as an indicator of peripheral adiposity [16], represents the muscle maintenance level and nutritional status of older people, and the association between CC and cognitive function seems to be closer than BMI [14, 15]. Therefore, to better understand the influence of obesity on cognitive function, it is necessary to distinguish the difference between muscle mass and fat. Low FFM or high FM are abnormal body composition phenotypes associated with morbidity. A potential method to assess the combined effect of FM and FFM is to express these variables using a ratio, FM/FFM [17]. Sarcopenic obesity as defined by FM/FFM is associated with cognitive impairment [17]. At present, there is still a lack of research on the association between CC, WCR, FM/FFM and cognitive impairment, especially the differences between different ages or sexes.

Therefore, this study used common obesity evaluation indicators (BMI, WC, WHR) and CC, WCR, FM/FFM to evaluate obesity, and analyzed the relationship between these indicators of obesity and cognitive impairment among suburb-dwelling older people in China. In addition, we also explored whether the relationship was maintained between different age or sex subgroups. Finally, we analyzed the relationship between obesity indicators and specific cognitive domains.

## Materials and methods

This was a cross-sectional study conducted from August 2018 to October 2020, using data from Adult Physical Fitness and Health Cohort Study (APFHCS) [ChiCTR1900024880]. APFHCS is a large prospective dynamic cohort study, mainly investigated the association between physical fitness and health status in a general adult population living in Tianjin and Shanghai, China. This study was approved by the Ethics Committee of Tianjin Medical University and Shanghai University of Medicine and Health Sciences, and participants provided written informed consent before participation.

### Study participants

The APDHCS survey collected a comprehensive collection of health testing indicators and evaluation systems of older people (≥ 60 years). Before the survey, we explained the consent process, study procedures, and purpose to the participants. The inclusion criteria were willingness to participate and cooperate with relevant inspections in the study. Exclusion criteria of this study were as follows: (1) severe disease that prevents independent mobility; (2) inability to stand for measurement or unable to stand for measurement of body composition, weight, and height; (3) unable to cooperate with the investigators.

A total of 2029 people residing in Hangu District, Tianjin and Chongming District, Shanghai, took part in the national free physical examination program as part of a comprehensive geriatric assessment. A total of 276 participants were excluded because of missing values or exclusion criteria, leaving 1753 available for analysis.

### Data collection

#### Assessment of cognitive function

Cognitive function was determined by the Mini-Mental State Examination (MMSE) which included 30 items. The maximum score of MMSE is 30, with higher scores indicating better cognitive function. Cognitive impairment was defined as a score of ≤ 17 for illiterates, ≤ 20 for participants with primary school education, and ≤ 24 for those with junior high school degrees or above [14]. Specifically, the MMSE includes a broad set of cognitive domains that measure the following: orientation to time (5 points), orientation to place (5 points), registration (3 points), attention and calculation (5 points), recall (3 points), and language (9 points) [18].

#### Anthropometric measurements

The core elements of anthropometric measurements in this study were height, weight, hip circumference, WC, and CC. Weight was measured with an electronic scale while participants were wearing indoor clothing and no shoes. Height to the nearest 0.5 cm was measured under the same conditions with a wall-mounted stadiometer. WC was measured at the minimal central perimeter located halfway between the rib cage and the pelvic crest. Hip circumference was measured at the point of maximal protrusion of the gluteal muscles and, in the anterior plane, the symphysis of the pubis [19]. CC was measured at the point of the largest circumference of the calf. The measurement of the WC, CC, and hip circumference were all measured with a flexible tape measure. During the operation, the tape was kept in close contact with the skin, and the subcutaneous tissues are not compressed. To avoid measurement error, postgraduate students in the health field who had received special training for testing administered all tests as part of a standardized geriatric assessment. BMI = weight (kg)/height^2^ (m^2^); Waist-to-hip ratio (WHR) = waist circumference(cm)/hip circumference(cm); WCR = waist circumference(cm)/calf circumference(cm).

#### Body composition measurements

Body composition characteristics were obtained using a bioelectrical impedance analyzer (Inbody720; Bio space Co., Ltd, Seoul, Korea). Assessments included fat-free mass and body fat mass. Participants were required to be dressed in light clothes, be barefoot, and tested for body composition 2–4 hours after breakfast in the morning for body composition measurements [20]. Details of survey methods had been described in our previous cross-sectional study [20].

#### Covariates

During the face-to-face evaluation, data associated with lifestyle factors was obtained by a standardized questionnaire and included age, sex, and education level (illiteracy, primary school, or middle school and above), living situation (living together with families and living alone), smoking habits (current smoker, never smoked, or former smoker), drinking habits (daily drinkers, occasional drinkers, former drinker, or never a drinker), marital status, nutrition, and depression. Marital status was classified as married (living together, divorced, separated, or widowed) or never married. Nutritional status was assessed on the Mini Nutritional Assessment Short-Form (MNA-SF) scale; a score of ≤ 11 was considered to malnutrition [21]. Depression was identified on the 30-item Geriatric Depression Scale (GDS-30) scale; a score of ≥ 11 was considered to indicate depression [22]. Data recorded also included a medical history and history of treatment (e.g. diabetes mellitus, hypertension, hyperlipidemia). These diseases were defined by self-reporting a physician’s diagnosis or the use of diagnosed medications. Physical activity level was assessed with the short form of the International Physical Activity Questionnaire (IPAQ) [23]. Responses were converted to metabolic equivalent task minutes per week (MET-min/week) through multiplying total minutes over the previous 7 days spent on vigorous activity, moderate-intensity activity, and walking by 8.0, 4.0, and 3.3 respectively and then summing to indicate overall physical activity [22, 23].

### Statistical analysis

Differences in characteristics were assessed by independent sample t-test for continuous variables, and by Chi-square tests for categorical variables between the participants with cognitive impairment and without. The data were presented as means ± SD or as numbers (percentages). Logistic regressions were used to examine the associations between obesity indicators and cognitive impairment; The obesity indicators in quartiles used the lowest quartile as the reference. All the analyses were repeated, stratified by sex and age group (≤ 70 years, 70 − 80 years, and ≥ 80 years) including the adjustments described for the final model. The status of each cognitive domain (recall, registration, orientation, language, attention, and calculation) and MMSE score was set as the dependent variable and the common obesity evaluation indicators (BMI, WC, WHR) as well as CC, WCR, and FM/FFM were set as the independent variable. Multivariable linear regression models were used for modeling the relationship between MMSE score, cognition domain, and obesity-related indicators. Restricted cubic splines were used to display and test the association between the cognitive impairment and obesity-related indicators.

For all regression analyses, fully adjusted models were adjusted for age, sex, education, marital status, living situation, smoking, drinking, physical activity, diabetes, hyperlipidemia, hypertension, stroke, nutrition, and depression. For the multiple logistic regression analysis, odds ratios (OR) and 95% confidential intervals (CI) were calculated. Statistical analysis was conducted by SPSS version 23.0. The significance level for all tests was set at a two-tailed *P* value of < 0.05. Restricted cubic splines were conducted by R 3.5.2.

## Results

### Characteristics of study participants

Table 1 provided the characteristics of the sample. Data of 1753 participants (41.0% men; aged 71.36 ± 5.96 years) was analyzed, and (329) 18.77% were classified as cognitively impaired by MMSE score. The MMSE scores of the cognitively impaired were significantly lower than that of the normal cognitive group (17.07 ± 4.03 score vs. 25.88 ± 2.90 scores). The cognitively impaired and those with normal cognition also differed significantly with respect to some anthropometric variables. The cognitively impaired had higher WCR, FM/FFM, and lower CC, fat-free mass when compared to the group with normal cognition (*P* all < 0.001). The participants with cognitive impairment tended to be older, female and had less physical activity, more prevalence of hypertension, and higher depression scores than cognitively normal participants. Additional factors that were associated with cognitive impairment included whether they were educated, and to what degree they were educated, and whether widowed and/or living alone.

**Table 1:** Characteristics of study group, stratified by cognitive status.

Variables	Normal Cognition	Cognitive impairment	Total	*P* − value
	(N = 1424)	(N = 329)	(N = 1753)	
Age (y)	70.81 ± 5.52	73.75 ± 7.12	71.36 ± 5.96	< 0.001
Sex, n (%)				< 0.001
Male	624 (43.8)	94 (28.6)	718 (41.0)	
Female	800 (56.2)	235 (71.4)	1035 (59.0)	
Anthropometric indicators				
BMI (kg/m^2^)	24.00 ± 3.44	23.99 ± 3.58	24.00 ± 3.47	0.949
WC (cm)	89.53 ± 9.73	89.16 ± 10.16	89.46 ± 9.81	0.537
CC (cm)	33.93 ± 3.01	32.81 ± 3.20	33.72 ± 3.08	< 0.001
WHR	0.92 ± 0.07	0.92 ± 0.06	0.92 ± 0.06	0.797
WCR	2.64 ± 0.26	2.72 ± 0.28	2.66 ± 0.71	< 0.001
Fat mass (kg)	17.20 ± 6.35	17.41 ± 6.61	17.24 ± 6.40	0.582
Fat-free mass (kg)	44.87 ± 8.31	41.47 ± 8.09	44.24 ± 8.37	< 0.001
FM/FFM	0.39 ± 0.15	0.42 ± 0.16	0.40 ± 0.15	< 0.001
Widowed, n (%)	218 (15.3)	105 (31.9)	323 (18.4)	< 0.001
Living alone, n (%)	188 (13.2)	81(24.6)	269 (15.4)	< 0.001
Education, n (%)				< 0.001
Illiteracy	175 (12.3)	114 (34.7)	289 (16.5)	
Primary school	856 (60.1)	147 (44.7)	1003 (57.2)	
Middle school and above	393 (27.6)	68 (20.7)	461 (26.3)	
Smoking, n (%)				0.087
Current smokers	249 (17.5)	55 (16.8)	304 (17.4)	
Never smokers	922 (64.9)	231 (70.4)	1153 (66.0)	
Ex-smokers	249 (17.5)	42 (12.8)	291(16.6)	
Drinking, n (%)				0.086
Daily drinkers	195 (13.9)	39 (12.1)	234 (13.6)	
Occasional drinkers	199 (14.2)	39 (12.1)	238 (13.8)	
Former drinkers	157 (11.2)	25 (7.8)	182 (10.6)	
Never drinkers	852 (60.7)	219 (68.0)	1071 (62.1)	
Chronic conditions, n (%)				
Hypercholesterolemia	198 (13.9)	36 (10.9)	234 (13.4)	0.343
Hypertension	800 (56.2)	212 (64.4)	1012 (57.8)	0.006
Diabetes	217 (15.3)	57 (17.3)	274 (15.7)	0.355
Stoke	91 (6.1)	27 (7.7)	118 (6.4)	0.276
IPAQ (Met/wk)	4746	3360	4519	< 0.001
	(2205, 8652)	(1533, 6951)	(2074, 8253)	
MNA-SF, score	12.66 ± 1.51	12.28 ± 1.67	12.59 ± 1.55	< 0.001
Depression, score	5.46 ± 4.32	7.00 ± 5.56	5.75 ± 4.61	< 0.001
MMSE, score	25.88 ± 2.90	17.07 ± 4.03	24.21 ± 4.65	< 0.001

Data are shown as mean ± SD or P 50 (P 25, P 75) or number (percentage); BMI: body mass index; WC, waist circumference; CC: calf circumference; WHR: waist to hip ratio; WCR: waist to calf circumstance ratio; FM/FFM: fat to fat-free mass; IPAQ: International Physical Activity Questionnaire; MET/wk, metabolic equivalent task minutes per week; MNA-SF: Mini-Nutritional Assessment-Short Form; MMSE: Mini-Mental State Examination.

### Association between obesity-related indicators and cognitive function

As shown in Table 2, obesity-related indicators including BMI, WC, CC, WHR, WCR, and FM/FFM were included as continuous independent variables, with cognitive impairment as the dependent variable. In the fully adjusted model, CC was negatively associated with cognitive impairment (OR = 0.94, 95% CI: 0.90 − 0.98). However, the relationship between BMI, WC, WHR, WCR, FM/FFM, and cognitive impairment was not significant.

**Table 2:** Logistic regression analyses of the association of BMI, WC, CC, WHR, WCR and FM/FFM with cognitive impairment.

Variables	Crude		Basic model †		Final model ‡	
	OR (95% CI)	*P*-value	OR (95% CI)	*P*-value	OR (95% CI)	*P*-value
BMI (kg/m^2^)	0.99 (0.96 − 1.03)	0.949	1.00 (0.97 − 1.04)	0.584	1.02 (0.98 − 1.07)	0.178
WC (cm)	0.99 (0.98 − 1.00)	0.537	0.99 (0.98 − 1.01)	0.808	0.99 (0.98 − 1.01)	0.777
CC (cm)	0.88 (0.85 − 0.92)	< 0.001	0.93 (0.89 − 0.97)	0.003	0.94 (0.90 − 0.98)	0.031
WHR (cm/cm)	1.60 (0.30 − 8.50)	0.797	0.83 (0.15 − 4.66)	0.714	1.00 (0.15 − 6.54)	0.813
WCR (cm/cm)	3.16 (2.09 − 4.78)	< 0.001	1.75 (1.12 − 2.74)	0.015	1.69 (1.05 − 2.73)	0.078
FM/FFM (kg/kg)	4.58 (2.10 − 9.95)	< 0.001	1.74 (0.65 − 4.60)	0.171	2.89 (0.97 − 8.61)	0.052

BMI: body mass index; WC, waist circumference; CC: calf circumference; WHR: waist to hip ratio; WCR: waist to calf circumstance ratio; FM/FFM: fat to fat-free mass;

†Adjusted for potential confounders including age and sex;

‡ Adjusted for age, sex, education, marital status, living situation, drinking, smoking, physical activity, hypercholesterolemia, hypertension, diabetes, stoke, nutrition and depression.

Table 3 presented further division of BMI, WC, CC, WHR, WCR, and FM/FFM by quartile and inclusion as categorical variables in the logistic regression models, which showed that after full adjustment, compared to the WC participants in the first quartile, the risk of cognitive impairment in the second quartile WC increased, and the odds ratio was 1.59 (95% CI: 1.09 − 2.33). When compared to CC in the first quartile, the risk of cognitive impairment with the CC in the fourth quartile was reduced by 36%, and the odds ratio was 0.63 (95% CI: 0.40 − 0.98). However, no linear correlation was found between WC and CC with cognitive impairment. After full adjustment, there was a linear correlation between cognitive impairment risk and FM/FFM (*P* for trend = 0.029).

**Table 3:** Logistic regression analyses of the association of BMI, WC, CC, WHR, WCR and FM/FFM quartiles with cognitive impairment.

Variables	Q1	Q2	Q3	Q4	*P* for trend
BMI (kg/m^2^)					
*n (%)*	443 (25.3)	429 (24.5)	444 (25.3)	437 (24.9)	
Crude	Ref	0.95 (0.68 − 1.33)	0.84 (0.60 − 1.18)	0.93 (0.66 − 1.30)	0.391
Adjusted model†	Ref	1.44 (0.95 − 2.16)	1.17 (0.75 − 1.81)	1.27 (0.81 − 1.98)	0.575
WC (cm)					
*n (%)*	473 (27.0)	464 (26.5)	397 (22.6)	419 (23.9)	
Crude	Ref	1.22 (0.88 − 1.68)	0.81(0.56 − 1.16)	1.17 (0.84 − 1.64)	0.877
Adjusted model†	Ref	1.59 (1.09 − 2.33)*	1.00 (0.65 − 1.55)	1.30 (0.86 − 1.97)	0.748
CC (cm)					
*n (%)*	573 (32.7)	457 (26.1)	375 (21.4)	348 (19.9)	
Crude	Ref	0.56 (0.41 − 0.77)*	0.57 (0.41 − 0.77)*	0.38 (0.26 − 0.55)*	0.111
Adjusted model†	Ref	0.67 (0.47 − 0.96)*	0.83 (0.56 − 1.22)	0.63 (0.40 − 0.98)*	0.271
WHR (cm/cm)					
*n (%)*	377 (21.5)	485 (27.7)	442 (25.2)	449 (25.6)	
Crude	Ref	0.97 (0.68 − 1.37)	1.05 (0.74 − 1.50)	1.02 (0.72 − 1.45)	0.508
Adjusted model†	Ref	1.12 (0.75 − 1.65)	1.23 (0.82 − 1.85)	0.97 (0.65 − 1.47)	0.974
WCR (cm/cm)					
*n (%)*	440 (25.1)	436 (24.9)	446 (25.4)	431 (24.6)	
Crude	Ref	1.35 (0.94 − 1.95)	1.40 (0.97 − 2.00)	2.07 (1.46 − 2.94)*	0.029
Adjusted model†	Ref	1.25 (0.84 − 1.87)	1.26 (0.84 − 1.89)	1.25 (0.83 − 1.87)	0.264
FM/FFM (kg/kg)					
*n (%)*	425 (24.2)	461 (26.3)	424 (24.2)	443 (25.3)	
Crude	Ref	1.04 (0.72 − 1.50)	1.43 (1.00 − 2.04)*	1.79 (1.27 − 2.53)*	0.044
Adjusted model†	Ref	1.11 (0.73 − 1.70)	1.36 (0.85 − 2.17)	1.44 (0.88 − 2.35)	0.029

BMI: body mass index; WC, waist circumference; CC: calf circumference; WHR: waist to hip ratio; WCR: waist to calf circumstance ratio; FM/FFM: fat to fat-free mass;

*p<0.05;

†Adjusted for potential confounders including age, sex, education, marital status, living situation, drinking, smoking, physical activity, hypercholesterolemia, hypertension, diabetes, stoke, nutrition and depression.

As shown in Table 4, when exploring the differences in sex and age group, there was a significant linear increased risk of cognitive impairment with FM/FFM in women (*P* for trend = 0.002) and the oldest-old group (age ≥ 80 years) (*P* for trend = 0.009) after full adjustment. In addition, similar findings were found between BMI and cognitive impairment in participants aged 70 − 80 (*P* for trend = 0.011) and ≥ 80 years old (*P* for trend = 0.013).

**Table 4:** Logistic regression analyses of the association of BMI, WC, CC, WHR, WCR and FM/FFM quartiles with cognitive impairment, by sex and age group.

Variables	Sex †		Age group †		
	Men	Women	60 −70 years	70 − 80 years	≥ 80 years
	(n = 718)	(n = 1035)	(n = 784)	(n = 776)	(n = 193)
BMI (kg/m^2^)					
Q1	Ref	Ref	Ref	Ref	Ref
Q2	1.18 (0.59 − 2.35)	1.70 (1.01 − 2.88)*	1.96 (0.99 − 3.86)	1.13 (0.58 − 2.20)	1.25 (0.46 − 3.39)
Q3	1.05 (0.49 − 2.24)	1.35 (0.78 − 2.34)	1.20 (0.58 − 2.50)	1.27 (0.63 − 2.54)	1.36 (0.46 − 4.07)
Q4	0.98 (0.44 − 2.18)	1.55 (0.89 − 2.69)	1.27 (0.59 − 2.69)	1.35 (0.66 − 2.76)	1.50 (0.48 − 4.69)
*P* for trend	0.784	0.331	0.957	0.011	0.013
WC (cm)					
Q1	Ref	Ref	Ref	Ref	Ref
Q2	2.57 (1.26 − 5.25)*	1.38 (0.87 − 2.19)	2.08 (1.11 − 3.90)*	1.15 (0.62 − 2.12)	2.42 (0.88 − 6.59)
Q3	1.66 (0.74 − 3.73)	0.85 (0.50 − 1.45)	1.17 (0.57 − 2.39)	1.01 (0.51 − 1.98)	1.05 (0.35 − 3.13)
Q4	1.61 (0.71 − 3.68)	1.27 (0.78 − 2.08)	1.19 (0.57 − 2.46)	1.34 (0.71 − 2.53)	1.49 (0.53 − 4.17)
*P* for trend	0.780	0.790	0.953	0.255	0.957
CC (cm)					
Q1	Ref	Ref	Ref	Ref	Ref
Q2	0.53 (0.25 − 1.09)	0.80 (0.53 − 1.20)	1.43 (0.79 − 2.57)	0.45 (0.25 − 0.81)*	0.30 (0.12 − 0.78)*
Q3	0.93 (0.47 − 1.84)	0.83 (0.51 − 1.35)	1.08 (0.56 − 2.08)	0.94 (0.53 − 1.69)	0.31 (0.09 − 0.97)*
Q4	0.60 (0.28 − 1.25)	0.64 (0.36 − 1.15)	0.82 (0.40 − 1.68)	0.71 (0.37 − 1.37)	0.24 (0.05 − 1.02)
*P* for trend	0.537	0.077	0.564	0.801	0.128
WHR (cm/cm)					
Q1	Ref	Ref	Ref	Ref	Ref
Q2	1.07 (0.51 − 2.21)	1.15 (0.72 − 1.84)	1.29 (0.70 − 2.38)	1.01 (0.53 − 1.90)	1.09 (0.38 − 3.14)
Q3	1.19 (0.55 − 2.55)	1.29 (0.79 − 2.11)	1.25 (0.65 − 2.41)	1.24 (0.66 − 2.35)	1.22 (0.38 − 3.90)
Q4	1.09 (0.50 − 2.34)	0.95 (0.58 − 1.56)	0.83 (0.40 − 1.71)	0.93 (0.48 − 1.78)	1.03 (0.39 − 2.75)
*P* for trend	0.355	0.703	0.706	0.974	0.717
WCR (cm/cm)					
Q1	Ref	Ref	Ref	Ref	Ref
Q2	1.27 (0.65 − 2.47)	1.29 (0.77 − 2.17)	0.97 (0.53 − 1.80)	1.58 (0.83 − 3.02)	3.38 (0.97 − 1.77)
Q3	1.16 (0.59 − 2.27)	1.34 (0.79 − 2.25)	1.11 (0.62 − 2.04)	1.53 (0.80 − 2.95)	1.13 (0.62 − 3.56)
Q4	1.88 (0.94 − 3.78)	1.12 (0.67 − 1.87)	0.88 (0.43 − 1.77)	1.50 (0.79 − 2.85)	2.15 (0.62 − 6.37)
*P* for trend	0.100	0.720	0.621	0.318	0.828
FM/FFM (kg/kg)					
Q1	Ref	Ref	Ref	Ref	Ref
Q2	1.03 (0.57 − 1.88)	1.19 (0.58 − 2.44)	1.20 (0.59 − 2.42)	0.80 (0.42 − 1.55)	2.08 (0.71 − 6.05)
Q3	1.53 (0.74 − 3.18)	1.42 (0.70 − 2.87)	1.85 (0.86 − 3.96)	0.71 (0.34 − 1.52)	2.92 (0.93 − 9.11)
Q4	0.96 (0.29 − 3.16)	1.65 (0.81 − 3.34)	1.33 (0.58 − 3.05)	0.94 (0.43 − 2.05)	5.06 (1.45 −17.69)*
*P* for trend	0.859	0.002	0.451	0.769	0.009

BMI: body mass index; WC, waist circumference; CC: calf circumference; WHR: waist to hip ratio; WCR: waist to calf circumstance ratio; FM/FFM: fat to fat-free mass;

*p < 0.05; Data in the table are adjusted odds ratio (OR) and 95% confidence intervals (CI, in parentheses);

† Adjusted for potential confounders including age, sex, education, marital status, living situation, drinking, smoking, physical activity, hypercholesterolemia, hypertension, diabetes, stoke, nutrition and depression.

Table 5 summarized the relationship between CC and specific cognitive domains. In the fully adjusted model, CC was positively associated with recall (β:0.027, 95%Cl: 0.006− 0.048, *P* = 0.013) and orientation to place (β:0.022, 95%Cl: 0.009 − 0.036, *P* = 0.001).

**Table 5:** Multivariate linear regression analysis of the association between calf circumference and cognitive domains.

Variables	Crude			Basic model †			Final model ‡		
	β	95%CI	*P*-value	β	95% CI	*P*-value	β	95% CI	*P*-value
MMSE score	0.264	0.194 − 0.333	< 0.001	0.112	0.041 − 0.183	0.002	0.059	-0.009 − 0.126	0.087
MMSE subscores									
Recall	0.042	0.023 − 0.060	< 0.001	0.032	0.012 − 0.051	0.001	0.027	0.006 − 0.048	0.013
Language	0.056	0.036 − 0.076	< 0.001	0.017	-0.004 − 0.038	0.110	-0.001	-0.021 − 0.018	0.898
Registration	0.009	0.001 − 0.018	0.043	0.001	-0.008 − 0.010	0.815	-0.001	-0.011 − 0.009	0.877
Orientation to time	0.040	0.023 − 0.057	< 0.001	0.012	-0.006 − 0.029	0.194	0.003	-0.016 − 0.021	0.784
Orientation to place	0.048	0.036 − 0.061	< 0.001	0.021	0.009 − 0.034	< 0.001	0.022	0.009 − 0.036	0.001
Attention and calculation	0.069	0.043 − 0.095	< 0.001	0.029	0.002 − 0.057	0.036	0.009	-0.019 − 0.037	0.532

β: Unstandardized Coefficient; CI: Confidence Intervals;

†Adjusted for potential confounders including age and sex;

‡ Adjusted for age, sex, education, marital status, living situation, drinking, smoking, physical activity, hypercholesterolemia, hypertension, diabetes, stoke, nutrition and depression.

### Restricted cubic splines

**Fig 1:**
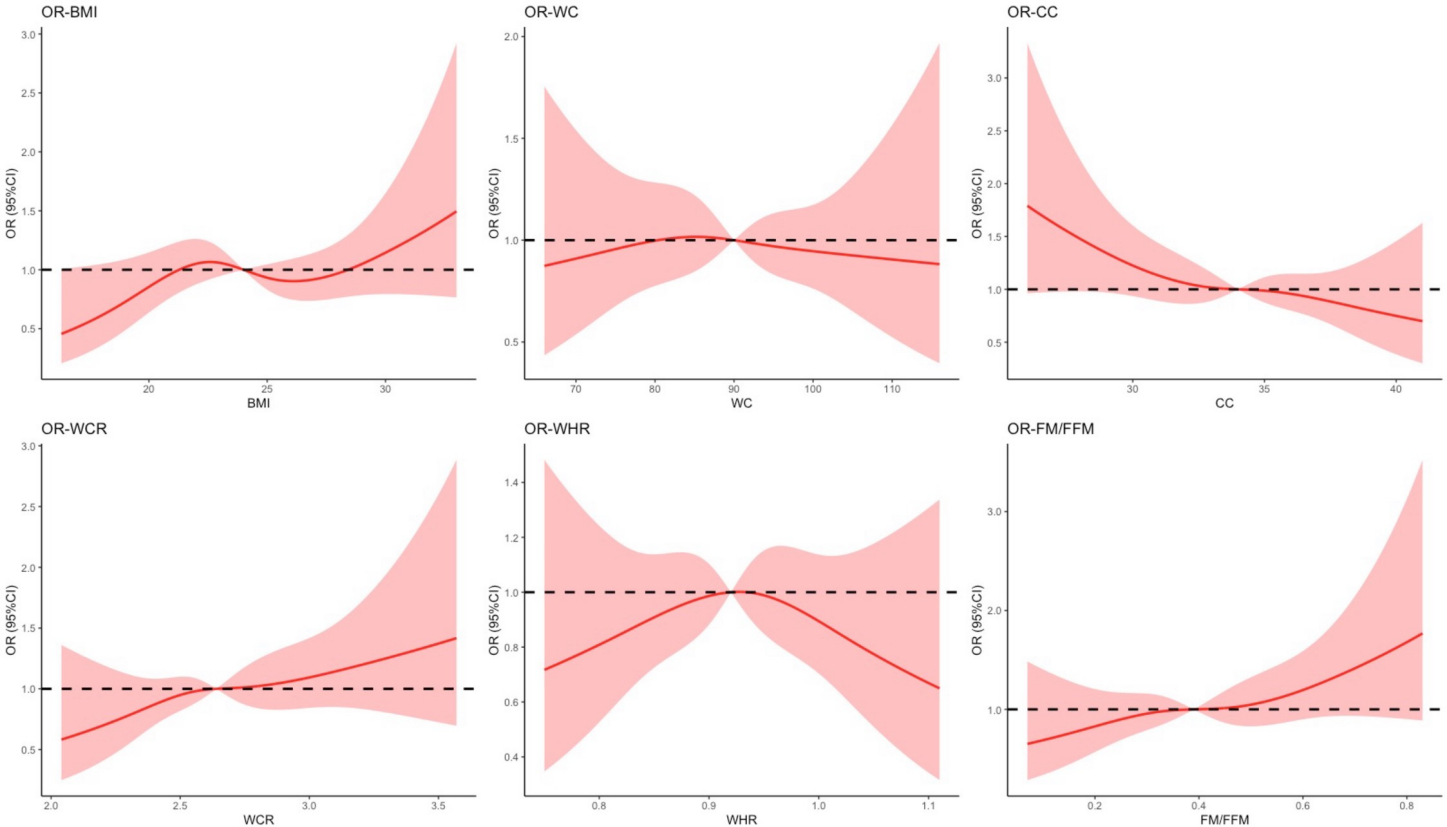
Restricted cubic splines assessing the association between obesity-related anthropometric indicators and cognitive function. Abbreviations: BMI: body mass index; WC, waist circumference; CC: calf circumference; WHR: waist to hip ratio; WCR: waist to calf circumstance ratio; FM/FFM: fat to fat-free mass. Age, sex, education, marital status, living situation, drinking, smoking, physical activity, hypercholesterolemia, hypertension, diabetes, stoke, nutrition and depression were adjusted for.

Restricted cubic splines were used to display and test the relationships between the obesity-related indicators (BMI, WC, CC, WHR, WCR, and FM/FFM) and cognitive impairment (Fig 1). With the increase of CC, the risks of cognitive impairment decreased (*P*_for nonlinearity_ = 0.749). With the increase of FM/FFM, the risks of cognitive impairment increased (*P*_for nonlinearity_ = 0.798).

## Discussion

This study was performed to understand the relationship between obesity-related indicators and cognitive impairment. The principal finding of this study was that CC was negatively associated with cognitive impairment. There was a linear correlation between cognitive impairment and FM/FFM in women and the oldest-old group, and so did the potential effect of BMI on cognitive function when the age >70 years old. To our knowledge, this study was the first study to incorporate FM/FFM into a measure of obesity and analyze its impact on cognitive function in suburb-dwelling older Chinese.

In our study, the prevalence of cognitive impairment using the MMSE was 18.77%. Women (22.7%) suffered from cognitive impairment more than men (13.1%). The average age of cognitive impairment was 73.75 ± 7.12 years (men: 73.08 ± 7.49 years, women: 74.02 ± 6.96 years). Previous studies of the Chinese population had shown a prevalence that was similar to that found in this study [24, 25].

Considering that abdominal fat and leg muscle mass have opposite effects on the risk of cognitive impairment, in addition to using WC and WHR to assess abdominal fat and CC to assess leg muscle mass, WCR was also used to assess the imbalance between abdominal fat and leg muscle mass [15]. An interesting finding in our study was that although there was no linear correlation between CC and the risk of cognitive impairment, CC may be a protective factor for cognitive impairment. This conclusion supported a study in Hainan, China, which suggested that CC was negatively associated with the risk of cognitive impairment [14]. Won et al. demonstrated that CC appeared as a significant predictor for multiple cognitive tests among both men and women [26]. Moreover, our study further found that CC associated positively with place orientation and recall. Studies have reported that when cognitive function is impaired, memory is the most affected [27]. The correlation between location orientation and calf circumference may be due to the important regulatory effect of vestibular reflex in the muscle group on neurocognitive function [28]. There is a link that CC represents subcutaneous fat which is considered a protective factor for cognitive function [29]. CC is a related parameter to evaluate the nutritional status of older people and a good indicator to measure the overall muscle mass, which means that among people with high CC, the brain changes related to cognitive impairment include medial temporal lobe atrophy and neuropathological burden is less [30–32]. Therefore, we pointed out that an assessment of CC may add value in a screening protocol for cognitive impairment risk in older adults, although a longitudinal follow-up is needed to determine whether higher CC prevents later-life cognitive impairment.

The risk of cognitive impairment increased linearly with the increase of FM/FFM, especially among women and the oldest. It should be noted that among all the obesity-related indicators, FM/FFM was most strongly associated with reduced MMSE total score in the fully-adjusted linear regression model (data was shown in S3 Table). This showed that the synergistic actions of fat mass and fat-free mass contributed to poorer cognition function. Our findings support an Asian study that reported sarcopenic obesity defined as FM/FFM > 0.80 was significantly associated with reduced cognitive impairment [17]. It is not clear what the exact pathophysiological mechanism is, but several explanations have been proposed. These include inflammation, oxidative damage, and insulin resistance [33, 34]. The sex differences are attributed to both a relatively higher fat mass proportion in women while a relatively higher fat-free mass proportion in men [35]. It was worth noting that the fat-free mass values were relatively lower among women and the oldest (data was shown in S1 and S2 Tables) and fat-free mass was negatively associated with cognitive impairment (data was shown in S4 Table). Therefore, we suggested that in assessing the impact of obesity on older people, attention should be paid to the distinction between fat mass and fat-free mass, especially the muscle mass of the lower extremities.

Notably, although the correlation between BMI and cognitive impairment did not appear, we found different results when we grouped people by age. Higher BMI was a risk factor for cognitive function when the age > 70 years old. Previous studies reported a complex relationship between BMI and cognitive function [3, 4, 10, 36, 37]. Although the cohort study reported in Lancet Diabetes Endocrinol showed the protective effect of higher BMI on cognitive function [10], the meta-analysis pooled data from 16 cohort studies did not support the beneficial impacts of overweight and obesity on incident dementia [3]. Another study proposed that the association between BMI and dementia differs depending on age at BMI measurement [37]. Therefore, results might be inconsistent due to methodological differences (e.g. follow-up duration, measure of cognition and obesity, or adjustments for confounding variables). The idea that we agreed with was the BMI-dementia association was attributable to two processes: a direct (causal) effect and reverse causation as a result of weight loss during the preclinical dementia phase [38].

Although previous studies have shown that WC, WHR [39, 40], and WCR [14] are negatively associated with cognitive impairment, no significant correlation has been found in our study. Our results are similar to those of Gardener and Waki [5, 6]. Although visceral fat is involved in insulin resistance and impairs cognitive function, subcutaneous fat is associated with lower insulin levels [29], high testosterone or estrogen levels are associated with higher cognitive function [41, 42], which brings cognitive advantages to obese people. In addition, with age, fat will be redistributed from the middle to the lower limbs. At the same time, our study differed from other studies in that our research objects were all suburban, had a history of work in high-intensity labor, and the economic level was relatively low. These findings might explain the lack of correlation between WC, WHR, and cognitive function in older people.

Overall, there is currently not enough evidence to explain the paradox of obesity in older adults. This study aimed to investigate the association between obesity-related indicators (specifically CC, WCR, and FM/FFM) and cognitive impairment, as better nutritional status and higher muscle retention are considered to be one of the mechanisms of the obesity paradox [15]. These indicators are easy to measure, risk-free, and convenient for clinical application. The recruitment groups in this study were special-suburban older men and women living in a discrete geographical area. They had a relatively active lifestyle but a low level of education, which may be different from participants in other geographical regions.

Several limitations should be considered in this study. First, our participants were relatively healthy and lack racial and economic diversity, so the study by default was restricted and the results might be biased. Second, one of the weaknesses of this study was the inaccuracy of body composition tools. Although we chose BIA as the measurement tool instead of dual-energy X-ray absorption (DXA), the two had proven to be well correlated. Furthermore, the design of the cross-sectional study showed only correlations rather than establishing causal relationships and might potentially suffer from reverse causations where those who were at risk of cognitive decline or dementia lose weight long before these events. More longitudinal research is needed while recruiting participants from different demographics.

## Conclusions

Our findings suggested that CC was negatively associated with cognitive impairment in older people. The risk of cognitive impairment increased linearly with the increase of FM/FFM, especially among women and the oldest, and so did the potential effect of BMI on cognitive function when the age > 70 years old. Future research needs to distinguish the effects of fat and muscle mass on cognitive function, with special attention to different ages and genders.

## Supporting information

S1 Table Mean of obesity-related indicators, stratified by cognitive status and age. (DOCX)

S2 Table Mean of obesity-related indicators, stratified by cognitive status and sex. (DOCX)

S3 Table Multivariate linear regression analysis of the association between obesity-related indicators and MMSE score. (DOCX)

S4 Table Logistic regression analyses of the association of FM and FFM with cognitive impairment. (DOCX)

S5 Table Correlation of calf circumference with other obesity-related indicators. (DOCX)
