# Supplementary material for: Relationship between obesity-related anthropometric indicators and cognitive function in Chinese suburb-dwelling older adults
Source: PLoS One. 2021 Oct 27;16(10):e0258922. doi: 10.1371/journal.pone.0258922 (PMC8550380; doi:10.1371/journal.pone.0258922)
Supplement: S1 Table — (DOCX) [file pone.0258922.s001.docx]

| **S 1 Table. Mean of obesity-related indicators, stratiﬁed by cognitive status and age.** | | | | | | | | | |
| --- | --- | --- | --- | --- | --- | --- | --- | --- | --- |
| **Variables** | **60 − 70 years** | | ***P*-value** | **70 − 80 years** | | ***P*-value** | **≥ 80 years** | | ***P*-value** |
|  | **Normal cognition**  **(n = 671)** | **Cognitive impairment**  **(n = 113)** |  | **Normal cognition**  **(n = 643)** | **Cognitive impairment**  **(n = 133)** |  | **Normal cognition**  **(n = 110)** | **Cognitive impairment**  **(n = 83)** |  |
| BMI (kg/m^2^) | 24.29 ± 3.29 | 24.26 ± 3.41 | 0.918 | 23.82 ± 3.52 | 24.34 ± 3.62 | 0.124 | 23.27 ± 3.70 | 23.05 ± 3.63 | 0.689 |
| WC (cm) | 89.57 ± 9.21 | 88.95 ± 9.42 | 0.508 | 89.47 ± 10.12 | 89.90 ± 10.11 | 0.661 | 89.61 ± 10.52 | 88.27 ± 11.18 | 0.394 |
| CC (cm) | 34.26 ± 2.90 | 33.90 ± 2.67 | 0.220 | 33.75 ± 3.11 | 33.02 ± 3.21 | 0.014 | 32.90 ± 2.85 | 30.97 ± 3.09 | < 0.001 |
| Fat mass (kg) | 17.82 ± 6.22 | 18.45 ± 6.82 | 0.329 | 16.74 ± 6.38 | 17.49 ± 6.62 | 0.218 | 16.06 ± 6.61 | 15.87 ± 6.08 | 0.841 |
| Fat-free mass (kg) | 45.86 ± 8.65 | 44.01 ± 7.23 | 0.032 | 44.17 ± 7.79 | 41.17 ± 7.91 | 0.001 | 42.91 ± 8.31 | 37.64 ± 8.09 | < 0.001 |
| WHR (cm/cm) | 0.91 ± 0.06 | 0.90 ± 0.05 | 0.294 | 0.92 ± 0.07 | 0.92 ± 0.06 | 0.638 | 0.93 ± 0.09 | 0.93 ± 0.08 | 0.635 |
| WCR (cm/cm) | 2.61 ± 0.25 | 2.62 ± 2.48 | 0.699 | 2.65 ± 0.27 | 2.73 ± 0.27 | 0.005 | 2.73 ± 2.92 | 2.85 ± 3.11 | 0.004 |
| FM/FFM (kg/kg) | 0.39 ± 0.15 | 0.42 ± 0.16 | 0.055 | 0.38 ± 0.15 | 0.42 ± 0.16 | 0.006 | 0.38 ± 0.15 | 0.43 ± 0.14 | 0.026 |
| *Note.* BMI: body mass index; WC, waist circumference; CC: calf circumference; WHR: waist to hip ratio; WCR: waist to calf circumstance ratio; FM/FFM: fat to fat-free mass. | | | | | | | | | |
