# Supplementary material for: Relationship between obesity-related anthropometric indicators and cognitive function in Chinese suburb-dwelling older adults
Source: PLoS One. 2021 Oct 27;16(10):e0258922. doi: 10.1371/journal.pone.0258922 (PMC8550380; doi:10.1371/journal.pone.0258922)
Supplement: S2 Table — (DOCX) [file pone.0258922.s002.docx]

| **S 2 Table** **.** **Mean of obesity-related indicators, stratiﬁed by cognitive status and sex.** | | | | | | |
| --- | --- | --- | --- | --- | --- | --- |
| **Variables** | **Man** | | ***P*-value** | **Woman** | | ***P*-value** |
|  | **Normal cognition**  **(n = 624)** | **Cognitive impairment**  **(n = 94)** |  | **Normal cognition**  **(n = 800)** | **Cognitive impairment**  **(n = 235)** |  |
| BMI (kg/m^2^) | 23.69 ± 3.30 | 23.63 ± 3.63 | 0.857 | 24.24 ± 3.53 | 24.13 ± 3.56 | 0.676 |
| WC (cm) | 90.00 ± 9.45 | 89.93 ± 10.12 | 0.944 | 89.16 ± 9.93 | 88.85 ± 10.18 | 0.675 |
| CC (cm) | 34.75 ± 2.90 | 33.86 ± 3.22 | 0.006 | 33.28 ± 2.95 | 32.38 ± 3.10 | < 0.001 |
| Fat mass (kg) | 15.22 ± 6.13 | 14.81 ± 6.34 | 0.544 | 18.74 ± 6.09 | 18.46 ± 6.44 | 0.537 |
| Fat-free mass (kg) | 51.48 ± 6.64 | 49.69 ± 7.22 | 0.016 | 39.71 ± 5.26 | 38.19 ± 5.74 | < 0.001 |
| WHR (cm/cm) | 0.92 ± 0.06 | 0.93 ± 0.06 | 0.621 | 0.91 ± 0.07 | 0.91 ± 0.06 | 0.784 |
| WCR (cm/cm) | 2.59 ± 0.24 | 2.66 ± 0.26 | 0.011 | 2.68 ± 0.27 | 2.75 ± 0.29 | < 0.001 |
| FM/FFM (kg/kg) | 0.29 ± 0.11 | 0.30 ± 0.12 | 0.623 | 0.46 ± 0.13 | 0.48 ± 0.14 | 0.261 |
| *Note.* BMI: body mass index; WC, waist circumference; CC: calf circumference; WHR: waist to hip ratio; WCR: waist to calf circumstance ratio; FM/FFM: fat to fat-free mass. | | | | | | |
