# Supplementary material for: Relationship between obesity-related anthropometric indicators and cognitive function in Chinese suburb-dwelling older adults
Source: PLoS One. 2021 Oct 27;16(10):e0258922. doi: 10.1371/journal.pone.0258922 (PMC8550380; doi:10.1371/journal.pone.0258922)
Supplement: S3 Table — (DOCX) [file pone.0258922.s003.docx]

| **S 3 Table .**  **Multivariate linear regression analysis of the association between obesity-related indicators and MMSE score.** | | | | | | | | | |
| --- | --- | --- | --- | --- | --- | --- | --- | --- | --- |
| **Variables** | **Crude** | | | **Basic model †** | | | **Final model ‡** | | |
|  | **β** | **95%CI** | ***P*-**  **value** | **β** | **95%CI** | ***P*-value** | **β** | **95%CI** | ***p*-value** |
| BMI (kg/m^2^) | -0.018 | -0.081 − 0.045 | 0.569 | -0.041 | -0.101 − 0.019 | 0.178 | -0.061 | -0.124 − 0.003 | 0.060 |
| WC (cm) | -0.007 | -0.029 − 0.015 | 0.547 | -0.014 | -0.035 − 0.007 | 0.203 | -0.009 | -0.030 − 0.012 | 0.400 |
| CC (cm) | 0.264 | 0.194 − 0.333 | < 0.001 | 0.112 | 0.041 − 0.183 | 0.002 | 0.059 | -0.009 − 0.126 | 0.087 |
| WHR (cm/cm) | -1.867 | -5.001 – 1.266 | 0.243 | -0.011 | -0.748 – 2.227 | 0.622 | -0.456 | -3.221 − 2.309 | 0.746 |
| WCR (cm/cm) | -3.128 | -3.917 − -2.338 | < 0.001 | -0.104 | -1.788 − -1.088 | 0.001 | -0.958 | -0.166 − -0.247 | 0.008 |
| FM/FFM(kg/kg) | -4.454 | -5.859 − -3.050 | < 0.001 | -1.735 | -3.360 – -1.111 | 0.036 | -1.750 | -3.335 − -0.166 | 0.030 |
| *Note.* BMI: body mass index; WC, waist circumference; CC: calf circumference; WHR: waist to hip ratio; WCR: waist to calf circumstance ratio; FM/FFM: fat to fat-free mass; β: unstandardized coeﬃcient; CI: confidence intervals. †Adjusted for potential confounders including age and sex; ‡ Adjusted for age, sex, education, marital status, living situation, drinking, smoking, physical activity, hypercholesterolemia, hypertension, diabetes, stoke, nutrition and depression. | | | | | | | | | |
