# Supplementary material for: Relationship between obesity-related anthropometric indicators and cognitive function in Chinese suburb-dwelling older adults
Source: PLoS One. 2021 Oct 27;16(10):e0258922. doi: 10.1371/journal.pone.0258922 (PMC8550380; doi:10.1371/journal.pone.0258922)
Supplement: S4 Table — (DOCX) [file pone.0258922.s004.docx]

| **S 4 Table. Logistic regression analyses of the association of FM and FFM with cognitive impairment.** | | | | | | |
| --- | --- | --- | --- | --- | --- | --- |
| **Variables** | **Crude** | | **Basic model †** | | **Final model ‡** | |
|  | **OR (95% CI)** | ***P*-value** | **OR (95% CI)** | ***P*-value** | **OR (95% CI)** | ***P*-value** |
| FM (kg) | 1.01(0.98 – 1.02) | 0.582 | 1.00 (0.98 – 1.02) | 0.823 | 1.01 (0.98 – 1.03) | 0.448 |
| FFM (kg) | 0.94 (0.93 – 0.96) | < 0.001 | 0.97 (0.95 – 0.99) | 0.040 | 0.97 (0.94 − 0.99) | 0.024 |
| Notes: FM: free mass; FFM: fat-free mass;†Adjusted for potential confounders including age and sex; ‡ Adjusted for age, sex, education, marital status, living situation, drinking, smoking, physical activity, hypercholesterolemia, hypertension, diabetes, stoke, nutrition and depression. | | | | | | |
