# Supplementary material for: Relationship between obesity-related anthropometric indicators and cognitive function in Chinese suburb-dwelling older adults
Source: PLoS One. 2021 Oct 27;16(10):e0258922. doi: 10.1371/journal.pone.0258922 (PMC8550380; doi:10.1371/journal.pone.0258922)
Supplement: S5 Table — (DOCX) [file pone.0258922.s005.docx]

| **S 5 Table** **. Correlation of calf circumference with other** **obesity-related indicators.** | | |
| --- | --- | --- |
| **Variables** | **r** | ***P*-value** |
| BMI (kg/m^2^) | 0.618 | < 0.001 |
| WC (cm) | 0.547 | < 0.001 |
| WHR (cm/cm) | 0.201 | < 0.001 |
| WCR (cm/cm) | -0.329 | < 0.001 |
| FM (kg) | 0.468 | < 0.001 |
| FFM (kg) | 0.569 | < 0.001 |
| FM/FFM (kg/kg) | 0.177 | < 0.001 |
| *Note.* BMI: body mass index; WC, waist circumference; CC: calf circumference; WHR: waist to hip ratio; WCR: waist to calf circumstance ratio; FM: free mass; FFM: fat-free mass; FM/FFM: fat to fat-free mass; | | |
